# Diabetic ketoacidosis complicated by emphysematous pyelonephritis: a case report and literature review

**DOI:** 10.1186/s12894-020-0575-0

**Published:** 2020-01-29

**Authors:** Yuanhao Song, Xingping Shen

**Affiliations:** Department of Endocrinology, Zhongshan Hospital Xiamen University, Xiamen, 361004 Fujian China

**Keywords:** Emphysematous pyelonephritis, Diabetes, Diabetic ketoacidosis, Kidney stones, Antibiotics

## Abstract

**Background:**

The management of emphysematous pyelonephritis (EPN) includes conservative medical treatment, percutaneous drainage, and surgical resection of the involved kidney. EPN with diabetic ketoacidosis(DKA) is very rare, in which the clinical management of refusing surgical drainage is inexperienced.

**Case presentation:**

A 34-year-old woman presented with abdominal pain, chills, fever, nausea, vomiting, chest tightness, and shortness of breath. Blood test results were consistent with diabetic ketoacidosis. Urinary computed tomography scan showed multiple stones in the right kidney and lower ureter, with right hydronephrosis. Blood culture demonstrated *Escherichia coli* bacteremia, and EPN was diagnosed. Considering the need for a second percutaneous nephrolithotomy, the patient refused percutaneous drainage. After continuous intravenous infusion of small doses of insulin and antibiotic treatment, the ketoacidosis resolved. The patient’s temperature returned to normal and abdominal pain was alleviated, and liver and kidney functions were also back to normal. After hospital discharge, the patient underwent two percutaneous nephrolithotomy in the department of urology.

**Conclusions:**

EPN with diabetic ketoacidosis should be diagnosed as soon as possible. For patients with Class 1 and Class 2 EPN with diabetic ketoacidosis and urinary tract obstruction, if surgical drainage is refused, it is particularly important to rapidly correct diabetic ketoacidosis and intravenous use of sensitive antibiotics, so as to create conditions for follow-up percutaneous nephrolithotomy.

## Background

Emphysematous pyelonephritis (EPN) is an acute, severe necrotizing infection affecting renal parenchyma, collecting system as well as surrounding tissue with hallmark of presence of gas within these structures [1]. Several large-scale retrospective studies have shown that affected patients commonly presented with fever, abdominal pain, nausea, and vomiting [1, 2]; although, occasionally, patients have experienced no obvious symptoms [3]. There has been no general consensus with respect to the diagnosis and treatment of EPN. A computed tomography (CT) scan is currently the gold-standard diagnostic test [4]. Treatment includes conservative medical therapy, with or without surgical drainage or nephrectomy [5].

At present, EPN with diabetic ketoacidosis is rare, only a few cases have been reported [6–10]. And diabetic ketoacidosis is an important predictor of death in patients with EPN [11]. Effective surgical drainage is a key measure for the management of EPN with diabetic ketoacidosis and urinary tract obstruction. In this case, the clinical management of patients refusing surgical drainage is inexperienced.

## Case presentation

A 34-year-old woman was admitted to the hospital due to abdominal pain, chills, fever, chest tightness, and shortness of breath after eating contaminated food 2 days earlier. After admission, she became nauseated and vomited; but without dysuria, urinary frequency, or urgency. She had a medical history of type I diabetes for 19 months, with poor control of blood glucose due to non-compliance with the insulin treatments. One month prior, she was found to have kidney stones. Physical examination revealed a temperature of 36.0 °C, pulse rate of 87 beats/minute, respiratory rate of 35 breaths/min, and a blood pressure of 105/66 mm of Hg. She had shortness of breath, dry skin, and dry mucous membranes and tongue; although her lungs sounded clear. There was an abdominal wall muscle strain in the epigastric area and right upper quadrant, with obvious tenderness. The right costovertebral angle also had tenderness.

**Fig. 1:**
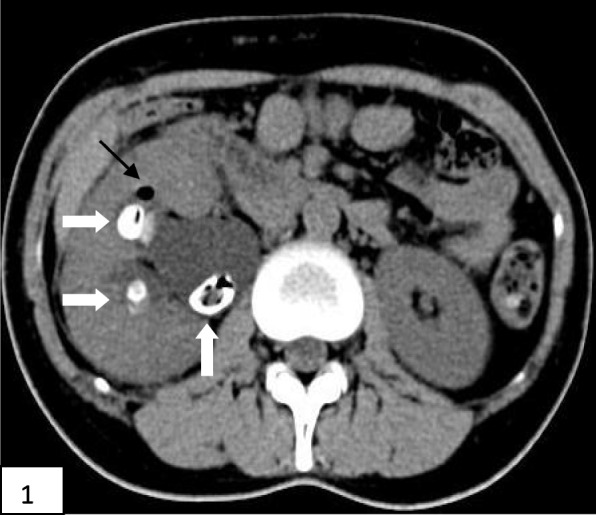
Show the non-contrast urinary CT scans during hospital admission

**Fig. 2:**
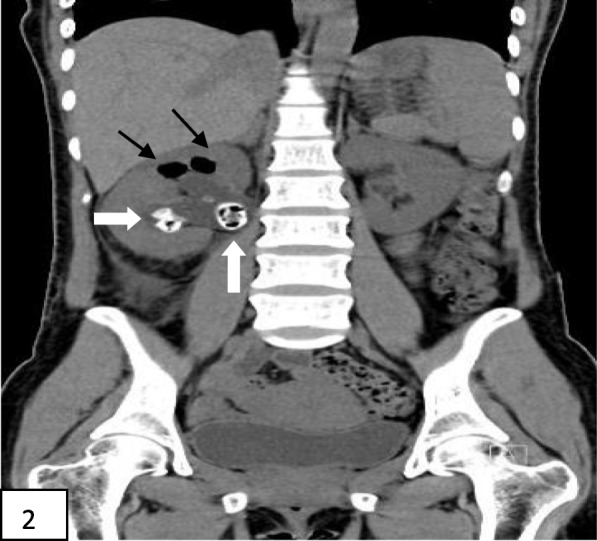
Show the non-contrast urinary CT scans during hospital admission

**Fig. 3:**
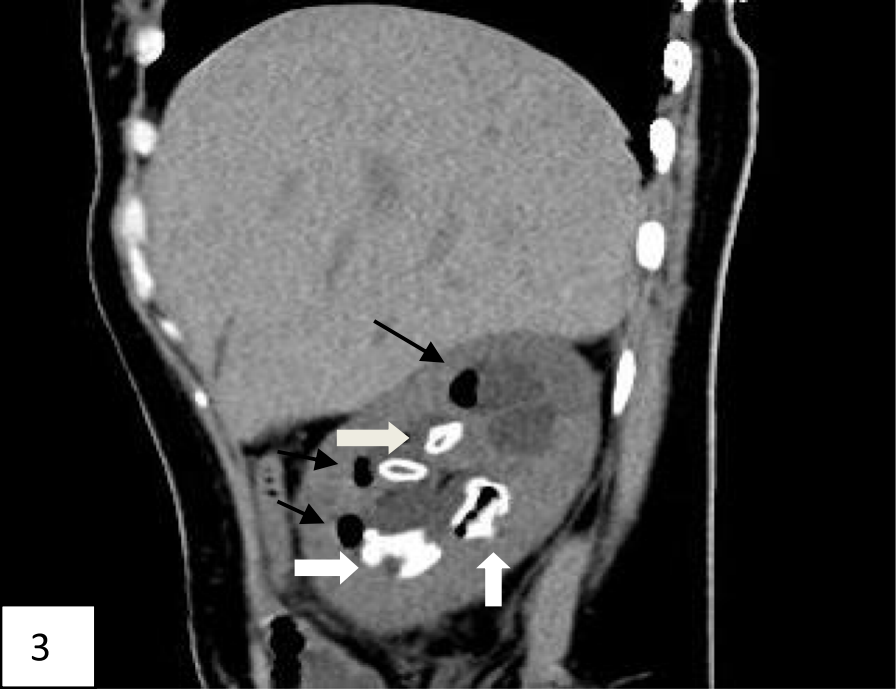
Show the non-contrast urinary CT scans during hospital admission

Laboratory tests and imaging studies were as follows. Blood tests after admission showed a white blood cell count of 21.08 × 10^9^/L, neutrophil count of 18.53 × 10^9^/L, neutrophil percentage of 87.9%, platelet count of 107 × 10^9^/L; and concentrations for albumin of 37.42 g/L, glucose of 35.95 mmol/L, urea of 10.54 mmol/L, creatinine of 145.1 μmol/L, bicarbonate of 2.2 mmol/L, potassium of 6.60 mmol/L, sodium of 122.10 mmol/L, β-hydroxybutyric acid of 10.08 mmol/L, lactic acid of 2.43 mmol/L, C-reactive protein of 448.39 mg/L, and procalcitonin > 100.000 ng/mL. Blood gas analysis showed a pH of 6.984, carbon dioxide partial pressure of 10.9 mm of Hg, oxygen partial pressure of 138.2 mm of Hg, measured bicarbonate of 2.5 mmol/L, calculated bicarbonate of 6.4 mmol/L, anion gap of 32.2 mmol/L, base excess of − 27.2 mmol/L, and carbon dioxide of 2.9 mmol/L. Urinalysis measurements were glucose, 4+; ketone body, 4+; leukocyte esterase, weakly positive; and white blood cell number, 41/μL; with negative results for urine culture. Blood culture identified *Escherichia coli*. Urinary CT scan revealed multiple stones in the right kidney and lower ureter, with right hydronephrosis; we therefore diagnosed EPN (Figs. 1, 2, and 3 reflect the CT scans during hospital admission).

**Fig. 4:**
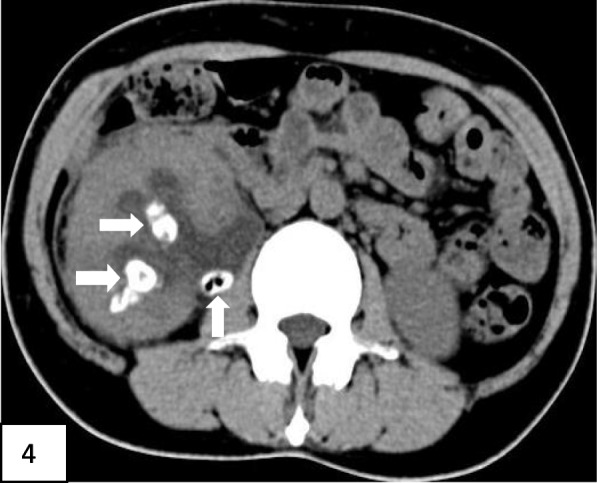
Shows the repeat non-contrast CT scan before hospital discharge

After being admitted to the hospital for definite diagnosis, our endocrinology department cooperated with urology, nephrology, infection department and imaging department to formulate a treatment plan, which suggested that the patients should undergo percutaneous drainage but the patient refused. Hospital management and outcomes after admission included intravenous infusion of small doses of insulin to correct ketoacidosis. Liver and renal functions returned to normal after albumin infusion and hydration. Based upon blood culture results, Cefoperazone sulbactam was upgraded to meropenem. Inflammatory indicators and body temperature gradually returned to normal, and abdominal pain was relieved. Repeated blood cultures were negative, and urine culture remained negative. Repeated urinary CT scans revealed multiple stones in the right kidney with worsening right hydronephrosis and pyelonephritis, but with reduced gas accumulation (Fig. 4).

**Fig. 5:**
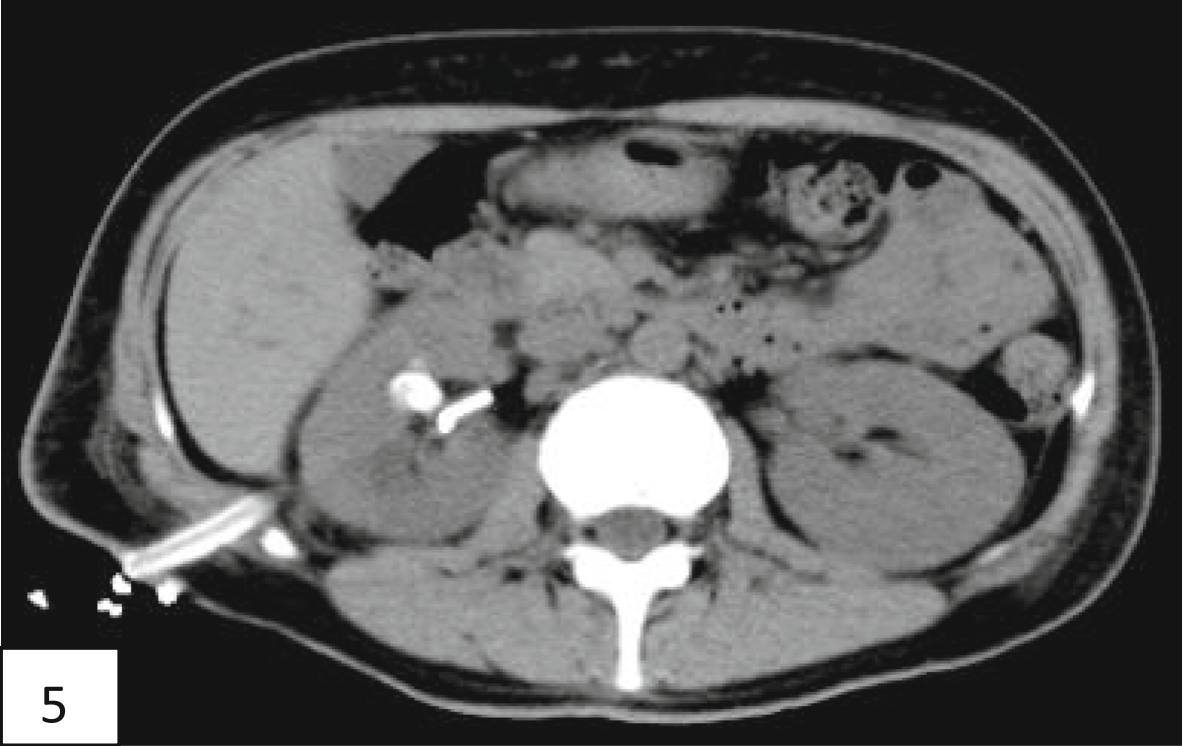
Shows the repeat non-contrast CT scan after the first percutaneous nephroscopy

**Fig. 6:**
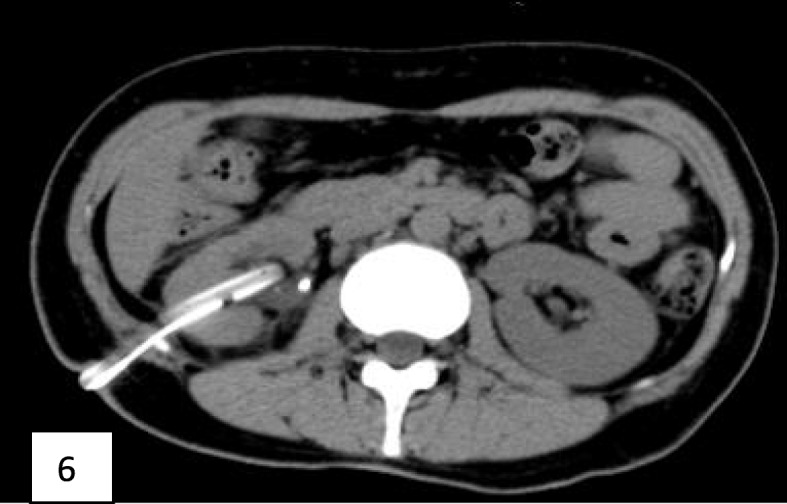
Shows the repeat non-contrast CT scan after the second percutaneous nephroscopy

One month after discharge, the patient underwent percutaneous nephrolithotomy in the department of urology, and the urinary CT was reexamined after operation (Fig. 5).Two months after discharge, the patient underwent the second percutaneous nephrolithotomy in the department of urology, and the urinary CT was reexamined after operation (Fig. 6). Currently, three months after discharge, clinical follow-up checks detect normal renal function and stable blood glucose control.

## Discussion and conclusions

In 1898, Kelly and MacCullum reported the first case of kidney infection with gas accumulation [12]. In 1962, Schultz and Klorfein described this disorder as emphysematous pyelonephritis [13]. Since then, there have been reports of various types of kidney infection with gas accumulation.

The pathogenesis of emphysematous pyelonephritis (EPN) is still unclear. It is believed that multiple factors, including diabetes, elevated glucose levels in kidney tissue, urinary tract obstruction, impaired renal circulation, decreased host immune function, and the presence of gas-producing microbial infections could cause EPN [1, 14]. EPN most commonly affects diabetic patients [2, 15–17]; and it is currently believed that the increased susceptibility to developing EPN in diabetic patients is due to impaired renal tissue perfusion [18]. Additionally, high blood glucose concentrations can also promote anaerobic growth and gas-producing metabolism [18].

CT scan is now considered to be the best method to diagnose EPN, and the most commonly accepted CT classification system for EPN was proposed by Huang and associates in 2000 [19]. These authors suggested 4 classes of EPN: Class 1, where gas is confined only to the collection; Class 2, where gas is located within the renal parenchyma and does not spread to the extrarenal space; Class 3A, where gas or an abscess spreads to the perinephric space; Class 3B, where gas or the abscess spreads to the pararenal space; and Class 4, depicting a bilateral or solitary kidney with EPN [19].. However, in one recent study, investigators attempted to re-correlate CT classification with clinical treatment options, analyzing 34 cases from 2009 to 2018. Their results showed that most Class 1 and Class 2 patients achieved satisfactory outcomes purely by conservative treatment. Only a small number of patients with urinary tract obstruction required combined percutaneous drainage, 2 of the 6 Class 3A patients required nephrectomy, and 2 Class 3B patients also required nephrectomy [16].

Lu et al. retrospectively analyzed 51 patients with EPN and demonstrated that the most common bacteria were *Escherichia coli*, followed by *Streptococcus pneumoniae*, *Enterococcus*, and *Pseudomonas aeruginosa*; with a small number of patients showing mixed bacterial infections. The antibiotic-resistance rate was greater than 15% for fluoroquinolone, ampicillin, 1st- and 2nd-generation cephalosporins, and gentamicin; whereas the resistance rates for the 3rd- and 4th-generation cephalosporins were only 10.9 and 6.5%, respectively. These authors concluded that the 3rd-generation cephalosporins could be used routinely for most affected patients. However, carbapenem antibiotics such as meropenem should be selected if a patient carries high risk factors, including recent hospitalization, antibiotic use, or DIC [20]. At the present time, most studies depict *Escherichia coli* as the most common pathogenic bacterium [1, 17]; although there have been recent reports of EPN caused by *Candida albicans* [21]. Therefore, selection of antibiotics should be based upon the local epidemiology and antibiotic- resistance patterns. In addition, multiple blood and urine cultures should be performed. It is extremely important to then revise the antibiotics according to the disease severity and the antibiotic-susceptibility test results.

There is recent evidence that patients who received conservative management achieved satisfactory results [15]. However, a study of 17 patients with EPN also showed that patients who received conservative treatment tend to experience acute increases in the Sequential Organ Failure Assessment (SOFA) score after the beginning of treatment, even if transient, which presents a great risk of septic mortality [15]. Adequate surgical drainage combined with intravenous antibiotics have resulted in satisfactory results in patients with EPN and multiple organ failure who were not appropriate candidates for nephrectomy [17, 22, 23]. In another study researchers tried to evaluate the clinical prognosis and outcomes of this disorder by analyzing 74 patients with EPN [1]. Their results showed that 1. fever was the most common clinical presentation, which was followed by lower back pain; 2. most patients had diabetes, which was followed by urolithiasis; 3. *Escherichia coli* was still the most common pathogen; 4. older age, high body-mass index, impaired renal function, thrombocytopenia, sensory changes, and shock were associated with a poor prognosis; and 5. treatment mainly consisted of rapid hydration, maintenance of electrolyte balance, use of systemic antibiotics, strict control of blood glucose, effective urine drainage, and nephrectomy if necessary [1]. Sanford et al. have shown that mild EPN was treatable by conservative management that included infectious agent-susceptible antibiotics [24]. However, patients with severe infections required percutaneous renal puncture drainage and/or double-J stenting [24].

Nanki et al. successfully treated a 58-year-old woman with EPN caused by *Escherichia coli* complicated with diabetic ketoacidosis through nephrectomy, antibiotics and recombinant human granulocyte colony-stimulating factor(rhG-CSF) [8]. Harrabi et al. reported a 64-year-old woman with EPN complicated with diabetic ketoacidosis who died of septic shock without surgical drainage [6]. However, another patient with EPN with diabetic ketoacidosis who underwent percutaneous renal drainage combined with antibiotics died 7 days after admission [9]; Unlike our patient, according to CT, this patient belongs to class 3A EPN. Recently, it has been reported that EPN with diabetic ketoacidosis in the patient with allogeneic renal transplantation improved after conservative treatment with intravenous antibiotics without percutaneous drainage or nephrectomy [10]; But our patient is different from this successful case in that there are urinary tract obstruction factors such as right kidney stone and right ureteral stone, and percutaneous drainage is more important in the treatment.

Our successful management of this patient lies in the following points: 1. We confirmed the diagnosis of EPN by urinary CT examination as soon as the patient was admitted to hospital; 2. Rapid correction of ketoacidosis by continuous intravenous infusion of low-dose insulin and fluid resuscitation; 3. We performed blood culture before using antibiotics and upgraded cefoperazone sulbactam to meropenem according to the results of blood culture and the antibiotic-susceptibility test results; 4. Our endocrinology department collaborated with urology, nephrology, infection and imaging departments to develop a treatment plan; 5. What is particularly important is that although the patient has sepsis, it belongs to Class 2 EPN. However, our treatment is also inadequate: the patient did not receive percutaneous drainage. Although the accumulation of gas in the kidney was less than before, the right hydronephrosis increased. In the end, the patient still underwent two percutaneous nephrolithotomy. Failed to achieve the goal that the patient wanted to have only one operation.

Our case report makes up for the gap in the clinical management of patients with EPN with diabetic ketoacidosis with urinary tract obstruction who refuse surgical drainage, but it is unclear whether our management experience can be extended to similar patients of EPN Class 3A,Class 3B and Class 4.

In conclusion, EPN with diabetic ketoacidosis should be diagnosed as soon as possible. For patients with Class 1 and Class 2 EPN with diabetic ketoacidosis and urinary tract obstruction, if surgical drainage is refused, it is particularly important to rapidly correct diabetic ketoacidosis and intravenous use of sensitive antibiotics, so as to create conditions for follow-up percutaneous nephrolithotomy.

## Data Availability

The datasets used and analysed during the current study are available from the corresponding author on reasonable request. All authors have read the paper and agree that it can be published.

## Abbreviations

CT: Computed tomography
DKA: Diabetic ketoacidosis
EPN: emphysematous pyelonephritis
RHG-CSF: Recombinant human granulocyte colony-stimulating factor
SOFA: Sequential Organ Failure Assessment
